# Case Report: Early presentation of hereditary angioedema symptoms in a 2-year-old boy

**DOI:** 10.3389/fped.2024.1408110

**Published:** 2024-06-24

**Authors:** Jurate Staikuniene-Kozonis, Juste Staikunaite, Edita Gasiuniene, Justina Sematonyte

**Affiliations:** ^1^ Department of Immunology and Allergology, Medical Academy, Lithuanian University of Health Sciences, Kaunas, Lithuania; ^2^ JSC CD8 Clinic, Kaunas, Lithuania; ^3^ Faculty of Medicine, Vilnius University, Vilnius, Lithuania

**Keywords:** hereditary angioedema (HAE), C1-INH deficiency, early onset hereditary angioedema, C1-INH-HAE, *SERPING1*

## Abstract

Hereditary angioedema (HAE) is a rare autosomal-dominant disease that is caused by a deficiency (type I) or dysfunction (type II) of the C1 inhibitor (C1-INH) due to a mutation in the *SERPING1* gene, which codes for C1-INH. HAE with quantitatively and qualitatively normal C1-INH (type III) is often caused by a mutation in the *F12* gene and no mutations in the *SERPING1* gene and is a group of very rare diseases. The C1 esterase inhibitor (C1-INH) is a major regulator of critical enzymes that are implicated in the cascades of bradykinin generation, which increases vascular permeability and allows the flow of fluids into the extracellular space, resulting in angioedema. HAE clinically manifests with intermittent attacks of swelling of the subcutaneous tissue or submucosal layers of the respiratory and gastrointestinal tract. Young children are typically asymptomatic, and those affected by HAE usually present with symptoms in their early 20s. This article describes the case of very early onset of hereditary angioedema caused by C1-INH deficiency in a 2-year-old boy who experienced recurrent episodes of hand and abdominal angioedema not associated with urticaria or pruritus. His father suffered from severe HAE due to a *de novo* mutation of the *SERPING1* gene. The same mutation of the *SERPING1* gene was detected in his son at the age of 9-months prior to the occurrence of angioedema symptoms, during genetic family counseling. This paper advances the understanding of HAE and highlights the importance of genetic counseling of families with HAE to avoid late or inaccurate diagnosis and to initiate treatment on time.

## 1 Introduction

Hereditary angioedema (HAE) is a rare autosomal-dominant disease that is caused by a deficiency or dysfunction of the C1 inhibitor (C1-INH) and affects 1/50,000 people worldwide (1–3). Three main types of HAE exist. Type I of HAE is characterized by a low level of C1-INH and type II (dysfunction of C1-INH) is caused by a mutation in the *SERPING1* gene that codes for the C1 inhibitor protein. Type III (quantitatively and qualitatively normal C1-INH) is often caused by a mutation in the *F12* gene and no mutations in the *SERPING1* gene. In this rare type of HAE, clinical manifestations are similar to those of C1-INH deficiency HAE, except that there is a higher female predominance due to aggravation during pregnancy and estrogen dependency (1–8). Currently, more than 700 different *SERPING1* variants are known to be linked to HAE type I and II (9). The pathophysiology of HAE is described as bradykinin-mediated. The C1 esterase inhibitor (C1-INH) is a regulator of the complement and contact systems and a major regulator of critical enzymes that are implicated in the cascades of bradykinin generation; such regulation increases vascular permeability and allows the flow of fluids into the extracellular space, resulting in angioedema. HAE clinically manifests with intermittent attacks of swelling of the subcutaneous tissue or submucosal layers of the respiratory or gastrointestinal tract (10). Attacks of swelling of tissues in the hands, feet, limbs, face, intestinal tract, or airway are triggered by precipitating factors such as emotional stress, trauma, hormonal influences, infections, and surgical or dental procedures (1–3). The severity and course of HAE may vary greatly even among family members harboring the same mutation (7, 8). The gene defect is already present at birth, but symptoms are uncommon in neonates and infants. Angioedema attacks can first occur at any age, but usually young children are asymptomatic, and those affected by HAE usually present with symptoms in their early 20s (3). In this study, we present the case of very early onset of hereditary angioedema caused by C1-INH deficiency in a 2-year-old boy who experienced recurrent episodes of angioedema of the extremities and face without urticaria and pruritus and an abdominal angioedema attack and also had a family history of HAE.

## 2 Case description

**Figure 1:**
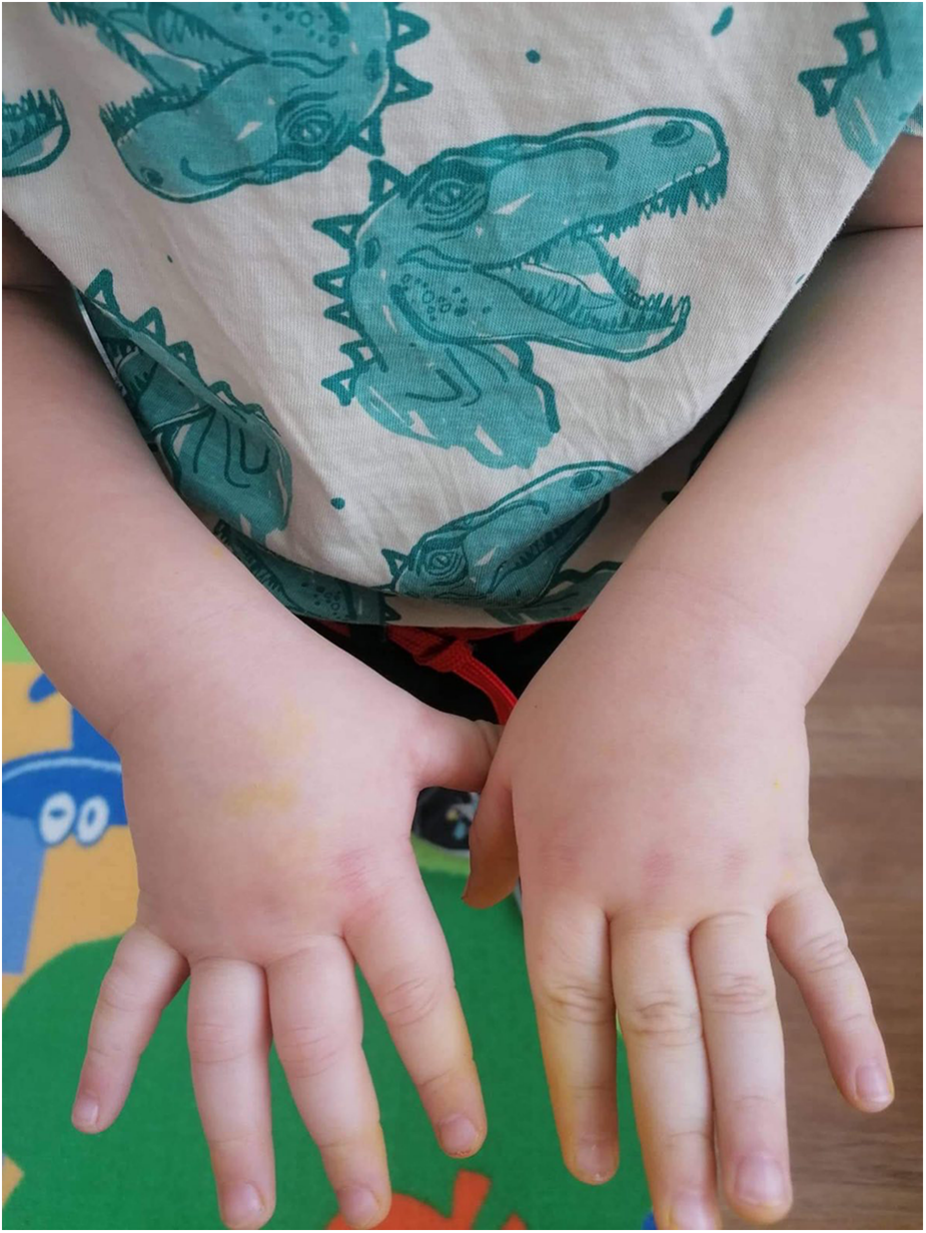
First attack of HAE angioedema of the hands (onset).

**Figure 2.**
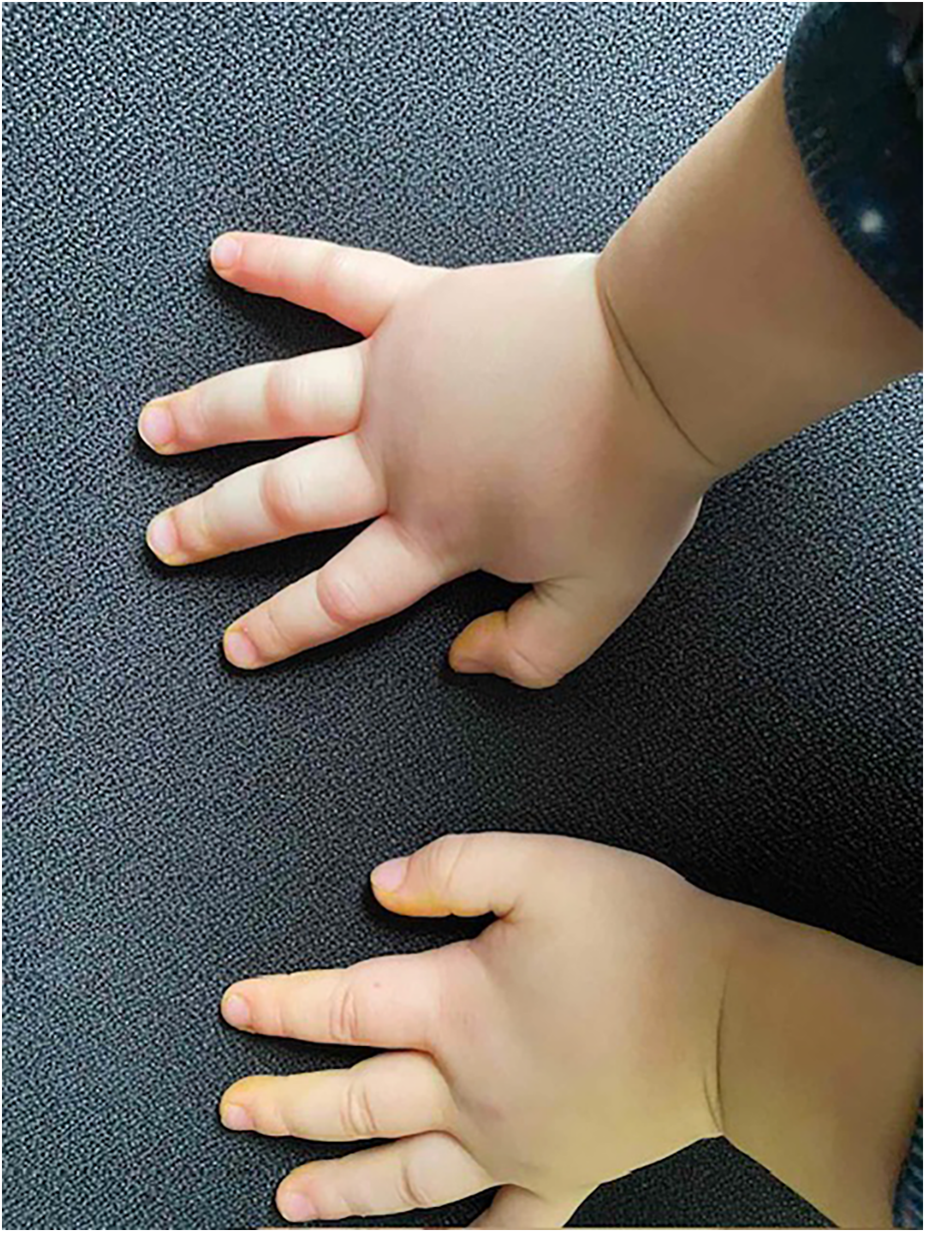
First attack of HAE angioedema of the hands (2 h later).

A 2-year-old boy started experiencing attacks of painful swelling in his face and upper extremities, which were triggered by a minor trauma and not associated with urticaria or pruritus. He was born normally from the first pregnancy and weighed 3,405 g at birth. The first episode of bilateral hand angioedema after the boy started playing with toys occurred at the age of 23 months and lasted 4 days (Figures 1, 2). The boy was immediately consulted by an allergologist-clinical immunologist and the serum complement component C4 and C1 inhibitor were evaluated, which revealed reduced levels of the following: C4 was 0.07 g/L (normal: 0.16–0.38), the C1 inhibitor was 0.05 g/L (normal: 0.15–0.35), and C3 in serum was 0.78 g/L (normal: 0.79–1.52) (Table 1). As the patient's father was suffering from HAE type I due to C1-INH deficiency and the same mutation of the *SERPING1* gene was detected in his son at the age of 9 months during genetic family counseling 1 year ago, the diagnosis of HAE type 1 due to C1-INH deficiency was established for this 2-year-old boy. HAE type I was diagnosed for the patient's father at the age of 23 years following recurrent peripheral and abdominal angioedema attacks with reduced C4 and C1-INH in serum. This diagnosis was made when he was consulted by an allergologist-clinical immunologist at the tertiary university hospital, and replacement therapy with C1-INH was initiated. For the father, it took 8 years from the onset of angioedema to confirm the diagnosis of HAE, before he was misdiagnosed as having systemic lupus, Crohn's disease, and appendicitis. Six years later when genetic testing was established in Lithuania, *de novo SERPING1* gene mutation was detected, and all of the boy’s family members, except his father who died at the age of 63 years from heart attack, were tested. The boy’s mother and sister had no mutations, but the same *SERPING1* gene heterozygous mutation (c.[473C>G]; [473=], p. [(Ser158Ter)]; [(Ser158=]) was detected in the boy, who was a 9-month-old asymptomatic child. At the time, this child was healthy with only a slightly reduced C1-INH level in serum (0.12 g/L, normal: 0.15–0.35 g/L). At the age of 2 years, the boy started experiencing angioedema attacks and treatment with plasma-derived C1 esterase inhibitor concentrate 500 IU (30 IU/kg) was initiated. At the follow-up of 2 years, it was found that the patient had experienced peripheral angioedema attacks only two times, but he had a history of one abdominal pain attack with vomiting that started 2 weeks after a common cold episode. In the emergency room, the abdominal ultrasound revealed terminal ileum wall thickness and a small amount of fluid in the pelvis and around the terminal ileum. Plasma-derived C1 esterase inhibitor concentrate 500 IU (30 IU/kg) was given intravenously for the angioedema attacks, following which symptoms resolved within 30 min. The patient was asked to continue the C1 inhibitor on-demand treatment.

**Table 1:** The complement system parameters of the patient and his father.

	Serum C1-INH level (g/L)	Serum C4 level (g/L)	Serum C3 level (g/L)
Before angioedema started (patient)	0.12	0.04	0.77
After the first angioedema attack (patient)	0.05	0.07	0.78
After angioedema attack (father)	0.09	0.04	0.83

Normal values in serum (g/L): C1-INH, 0.15–0.35; C4, 0.16–0.38; C3, 0.79–1.52.

## 3 Discussion

We present the case of very early onset of hereditary angioedema caused by C1-INH deficiency in a 2-year-old boy who experienced recurrent episodes of angioedema of the extremities and face without urticaria and pruritus and later an abdominal angioedema attack and also had a family history of HAE I. Our case is similar to the ones described in the literature, which shows that most attacks, and most first attacks, in children manifest with angioedema of the skin, and the earlier the onset of symptoms, the more severe the subsequent course of HAE I/II (1, 2). For C1-INH-HAE, the autosomal-dominant mode of inheritance has been described as that which occurs when one of the two alleles of the C1-INH gene (*SERPING1*) that encodes C1-INH is mutated. Mutations in *SERPING1* are responsible for the majority of cases of hereditary angioedema type I and II (6–9). The genotyping of subjects suffering from HAE has become diagnostically indispensable in clinical practice (11). In our presented case, the same *SERPING1* gene heterozygous mutation of one amino acid was confirmed for both the patient and his father, who had *de novo SERPING1* gene mutation. In the literature, it has been shown that only patients carrying missense mutations leading to a change in a single amino acid exhibited a less severe clinical phenotype; however, in our study, the boy started experiencing HAE symptoms at a relatively early age of 2 years when compared with his father, who had these symptoms when he was 15 years old. It is probable that there is no correlation between the same type of mutations and clinical phenotype. The involvement of epigenetic changes and environmental factors (i.e., temperature, pH, and oxidative stress) in the pathogenesis of HAE can also be postulated (6–8).

The detection of *SERPING1* mutation in a 9-month-old asymptomatic boy makes a strong argument for an early and timely diagnosis of HAE before peripheral angioedema attacks start at the age of 23 months, excluding other diseases and allergic angioedema. Apart from delayed diagnosis, misdiagnosis leads to inappropriate treatment, and by the time patients realize that they are misdiagnosed and want to make amends, it becomes too late, resulting in the denial of timely, , effective, and lifesaving treatment for them. However, in the presented case, early detection of *SERPING1* gene mutation and a family history of HAE prevented misdiagnosis and shortened the time to HAE diagnosis. From a large database of patients, HAE patients with family members diagnosed as having C1-INH-HAE were significantly less likely to be misdiagnosed than patients without a family history. Approximately 50% of patients with C1-INH-HAE type I or II have previously had their conditions misdiagnosed, most commonly as allergic angioedema or appendicitis (as was the case of our patient's father, when the HAE diagnosis was delayed for 8 years) (12–14). Therefore, we highly recommend that genetic family counseling be made standard care for patients affected by HAE.

Early diagnosis and appropriate treatment are essential for improving the lives of patients with this life-threatening disorder and disabling disease. HAE attacks of the upper airways can result in asphyxiation, abdominal attacks are painful and debilitating, and peripheral attacks of the hand and feet result in impaired function (1). The ultimate goals of treatment in HAE are to achieve total control of the occurrence of symptoms, which will reduce the frequency and severity of attacks, and to normalize patient lives (12). During the course of the Icatibant Outcome Survey (international, prospective, and observational registry collecting demographics and clinical outcomes in patients with HAE type I/II angioedema), triggers were identified in 56.0% of angioedema attacks, with the most common triggers being emotional distress, physical trauma, and infection (13). In the presented case, the 2-year-old boy experienced HAE attacks and these were associated with minor hand trauma while playing with toys and infection in case of abdominal attacks. During the course of the above-mentioned survey, the most commonly reported prodromal symptoms associated with attacks were erythema marginatum (13.2%), nausea (9.3%), irritability (7.3%), and change in estrogen levels in women (7.1%) (12). Probably because of the boy’s young age, prodromal symptoms were not observed in him.

Disease management for patients with HAE is currently achieved through the use of on-demand medications and short- and long-term prophylaxis. HAE attacks should be treated as early as possible and prophylaxis should be considered before known triggering events to reduce morbidity and mortality (1, 2). Acute treatment options include C1-INH replacement therapy [plasma-derived or recombinant human C1-INH (rhC1-INH) via intravenous administration], the kallikrein inhibitor ecallantide (subcutaneous administration, not approved for use in the EU including Lithuania, but approved in the United States), and the bradykinin B2 receptor antagonist icatibant (subcutaneous administration, not available in Lithuania). Short-term prophylaxis may be indicated before known triggers of swelling such as surgical or dental procedures. The available options include intravenous plasma-derived C1-INH (pdC1-INH), fresh frozen plasma, and attenuated androgens (e.g., danazol, oxandrolone). For long-term prophylaxis, there are options to include C1-INH (via intravenous or subcutaneous administration); subcutaneous lanadelumab, the fully human monoclonal antibody (mAb) against plasma kallikrein; attenuated androgens; and antifibrinolytics (tranexamic acid) (1, 2, 5, 14, 15). For children under the age of 12 years, the updated Lithuanian and international guidelines, including the World Allergy Organization (WAO)/European Academy of Allergy and Clinical Immunology (EAACI), recommend intravenous plasma-derived C1-INH and icatibant. Both are effective, well tolerated, and show a good safety profile (1, 16). The intravenous plasma-derived C1-INH concentrate given for the child in our study when he suffered acute angioedema attacks was the treatment of choice according to the guidelines, availability, and the fact of it being the only reimbursement option in Lithuania (16). For abdominal attacks, parenteral fluid replacement may be required as children are more susceptible to hypovolemia and dehydration, and extravasation into the peritoneal cavity and intestinal lumen can be substantial (1, 14, 15).

## 4 Conclusion

Our presented case is an original case report and strongly suggests that hereditary angioedema caused by C1-INH deficiency can occur very early in life. Early diagnosis and appropriate treatment are essential to improve the lives of patients with this disabling disease. As this disease is inherited in an autosomal-dominant way, the importance of genetic counseling of families with HAE is highly recommended to avoid late or inaccurate diagnosis of HAE and to initiate timely treatment to the affected members.

## Data Availability

The raw data supporting the conclusions of this article will be made available by the authors without undue reservation.
